# Ethically aligning a cardiovascular AI-DSS to exclude coronary artery disease with the clinical environment: a qualitative study among prospective end users

**DOI:** 10.1136/bmjdh-2026-000024

**Published:** 2026-10-20

**Authors:** Jojanneke Drogt, Karin Jongsma, Marieke Bluemink, Saskia Haitjema

**Affiliations:** 1 Bioethics and Health Humanities, University Medical Center Utrecht, Utrecht, The Netherlands; 2 Bartimeus Sonneheerdt Association, Zeist, The Netherlands; 3 Central Diagnostic Laboratory, University Medical Center Utrecht, Utrecht, The Netherlands

**Keywords:** Artificial intelligence, Medical Informatics, Decision Making, Computer-Assisted, Patient Care, Delivery of Health Care

## Abstract

**Objective:**

Perspectives and experiences of potential end users are essential to facilitate the ethical implementation of medical Artificial Intelligence (AI) models. In this study, we explored clinician and patient perspectives on the use and ethically relevant aspects of an AI-Decision Support System (AI-DSS), the ARGUS algorithm, developed for the exclusion of Coronary Artery Disease (CAD).

**Methods and analysis:**

We performed a qualitative study with semi-structured interviews to explore the perspectives of end users regarding the ARGUS AI-DSS. Cardiologists (n=11) and cardiology patients (n=20) were interviewed to study their expectations of this AI-DSS, the conditions under which they trust the outcome of the system and under which conditions they are willing to use it. All interview recordings were transcribed verbatim and a thematic analysis was conducted on the transcribed data.

**Results:**

Respondents in our study generally recognised the added value of using an AI-DSS targeted at excluding CAD as a supplement to clinical judgement. When discussing the anticipated benefits, both cardiologists and patients seemed to prioritise increased accuracy and efficiency while easing the patient burden was mentioned less frequently. One key concern raised by many cardiologists and patients—particularly aimed at AI systems focused on disease exclusion—is whether a low likelihood of CAD provides sufficient reassurance to patients; many may still choose to undergo a scan regardless of the algorithm’s recommendation. Further, respondents mentioned that the AI-DSS can lead to responsibility and trust shifts in clinical practice that are essential to consider before and during use.

**Conclusion:**

As disease exclusion becomes an emerging focus in AI development, the findings of this study offer key insights into how these can be successfully and ethically integrated into clinical practice. Additionally, the results provide important empirical insights that are relevant for ongoing ethical discussions regarding medical AI, namely: (1) the need for sex-specific AI-DSSs in cardiology, (2) shared responsibility for AI in clinical practice and (3) overruling the AI system in cases of human-AI disagreement.

**WHAT IS ALREADY KNOWN ON THIS TOPIC**

- While much attention has been drawn to the technical feasibility and performance of AI models, successful implementation also demands such systems to add value to clinical care. To ensure that AI-driven applications can be meaningfully integrated in clinical practice, in line with ethical values, the perspectives and experiences of potential end users are essential.

**WHAT THIS STUDY ADDS**

- Both cardiologists and patients seemed to prioritise increased accuracy and efficiency when considering benefits of exclusion-focused AI-DSS, while a relief of the patient burden was mentioned less frequently. A key concern raised by many cardiologists and patients—particularly aimed at AI systems focused on disease exclusion—is whether a low likelihood of CAD provides sufficient reassurance to patients; many may still choose to undergo a scan, regardless of the algorithm’s recommendation.
- This study provides an illustration of why it is necessary to resolve ethical unclarity about the need for sex-specific AI models in cardiology, how and who can bear responsibility for AI models in clinical practice and how to deal with human-AI disagreement.

**HOW THIS STUDY MIGHT AFFECT RESEARCH, PRACTICE OR POLICY**

- The interviews revealed a strong connection between this use case and broader unresolved questions in the ethical literature on AI, providing valuable insights into how AI ethics discussions apply to and are made specific in the clinical application of specific AI systems.
- As the study shows that ethical implementation is intertwined with its development, our research corroborates the importance of multidisciplinary AI development teams and reflecting on the ethical dimension of design choices early on.

## Introduction

 The significance of Artificial Intelligence (AI) for cardiovascular care is increasing.^1^ AI-based models for diagnosis are foreseen to benefit cardiovascular healthcare in terms of both efficacy and accuracy.^2–4^ While much attention has been drawn to the technical feasibility and performance of such models, successful implementation also demands systems to add value to clinical care. A prerequisite for their value creation is user adoption of the models in clinical care through applications such as websites, smartphone apps or AI-Decision Support Systems (DSS) that provide actionable insights. This means that aside from technical performance, attention should be paid to conditions for clinical implementation, surplus value for prospective end users, as well as the fit with the existing healthcare system. To ensure that AI-driven applications can be meaningfully implemented in line with ethical values, the perspectives and experiences of potential end users are essential. These shed light on the fit of an AI-DSS with the clinical environment, as their views support the evaluation of the ‘usefulness [of applications] for clinical practice and effects and impacts on clinical work, care processes and patient outcomes’.^5^ By making explicit what end users find important and what their values are, end-user perspectives can also help to identify ethically relevant aspects when these arise and provide insight into the practical applicability of ethical guidance.^6^ Their involvement can even increase their willingness to use AI systems, particularly if the systems are co-developed, and trust is fostered throughout the development process.^7^ The relevance of looking beyond the technological side of AI systems and focusing on social^8^ and practical ethical^9,10^ dimensions during development and implementation is widely argued for. To map the socioethical impact of medical AI applications, both qualitative research into stakeholders’ experiences and expectations^11^ and AI ethics research and guidelines^12^ have surged. A broad range of barriers and facilitators to the successful implementation of AI in clinical practice have been formulated,^13–16^ as has targeted and practice-oriented guidance regarding ethical considerations in the design and integration of AI.^17–19^ This has shed light on the importance of focusing on values such as fairness, responsibility, trust and meaningful oversight when considering the use of AI systems.^20^ At the same time, further research is required to tailor applications to the clinical context in which they would function and to determine how development can effectively bridge the gap between research and practice. Collaborative efforts among stakeholders in AI development, along with a focus on specific use cases,^21^ can help clarify how to align socioethical and technological aspects in concrete clinical settings.

To further investigate how specific systems can transition from research settings to practice and identify which socioethical considerations are important during the transition, we examine the compatibility of an AI-DSS developed within the clinical environment in which it is intended to operate and focus on the ethical aspects that warrant further analysis. The study is targeted at the ARGUS algorithm, which is developed and validated to exclude obstructive and non-obstructive Coronary Artery Disease (CAD) on the basis of routine electronic health record data in patients presenting with thoracic complaints.^22,23^ We interviewed cardiologists and cardiology patients, who are the prospective end users of this algorithm. Gaining better insight into their perspectives is particularly important as patient and clinician needs are often overlooked in AI development in cardiology, and clinician and patient perspectives are rarely combined.^24^ The insights gathered from this study are both novel and pertinent to the implementation of other AI-DSSs for several reasons. First, it highlights the potential benefits of AI-DSSs that focus on excluding diseases rather than prioritising various treatment options. While most AI-DSSs are designed to prioritise, theoretically, disease-exclusion models are a valuable addition as these could lead to more efficient use of limited resources, prevent unnecessary expenses for diagnostic tests and reduce patient burden.^25^ Second, implementing this specific AI-DSS prompts questions that are widely debated in the literature on AI ethics and thus provides a concrete example of how general ethical concerns play a role in the adoption of potential AI models. As mentioned in our discussion, it raises concerns regarding the need for sex-specific AI-DSS in cardiology, the concept of shared responsibility and how to deal with human-AI disagreement.

## Methods

### Study design

A qualitative study with semi-structured interviews was conducted at the University Medical Center Utrecht (UMCU), the Netherlands. The study is reported in accordance with the Consolidated Criteria for Reporting Qualitative Studies (COREQ).^26^ This empirical ethics study followed a reflexive thematic analysis approach. Further, our analysis is informed by the National Implementation Research Network (NIRN) Hexagon Model, with particular attention to the ‘need’ and ‘fit’ dimensions.^27^ These aspects were approached from an ethical perspective to support the interpretation of how participants evaluated the relevance, appropriateness and contextual alignment of the ARGUS tool. The focus on need and fit was considered particularly appropriate given the study context, as the integration of a sex-specific exclusion AI-DSS in cardiology is ethically sensitive and requires careful consideration of the system’s alignment with needs and values in clinical practice.^28,29^ This study constitutes part of the ARGUS study.^25^ Both patients (referred to as PT) as well as cardiologists (referred to as HCP, short for healthcare professional) were interviewed.

### Recruitment

Semi-structured in-depth interviews were conducted, with participants of the overarching study, after an email invitation. 31 participants were recruited from this sample, none of the approached people refused to participate (patients, n=20, and cardiologists, n=11; table 1). The topic list was designed to include the motivations, experiences and perspectives of the prospective end users. The semi-structured format provided participants with the opportunity to discuss matters they believed needed emphasising while offering guidance throughout the interview. Interviews were conducted by MB, a female researcher with experience in qualitative research. No relationship with participants was established prior to the interviews. Patients who met the following inclusion criteria were included through purposive sampling: participant in ARGUS study,^25^ able to speak either Dutch or English and having access to a computer (or another device) with internet access, camera and microphone. We aimed for an equal distribution of women and men.

**Table 1:** Background characteristics participants

Patients (n=20)		
Age	Mean±SD	60,30±7444
Gender	Number (%)	
Male		9 (45%)
Female		11 (55%)
Level of education	Number (%)	
Mid-level secondary school		2 (10%)
Senior secondary vocational education		5 (25%)
Higher vocational education		6 (30%)
Academic propaedeutic certificate		1 (5%)
Academic education		6 (30%)
Healthcare professionals (n=11)		
Gender	Number (%)	
Male		7 (64%)
Female		4 (36%)
Profession	Number (%)	
Cardiologist		9 (82%)
Cardiology fellow		2 (18%)
Centre type	Number (%)	
Academic		4 (36%)
Peripheral		7 (64%)

Healthcare professionals were personally approached or could register in response to a call at the Working group Cardiology centers Netherlands (WCN) forum. Healthcare professionals had to meet the following selection criteria: employed at a Dutch hospital, working as a cardiologist or cardiology fellow and having access to a computer (or another device) with internet access, camera and microphone. We aimed for the inclusion of both cardiologists working at tertiary referral centres as well as peripheral hospitals.

### Data collection

The interviews were conducted online (because of COVID-19 restrictions) between September 2020 and May 2021. After verbal informed consent, interviews were held via Webex or Microsoft Teams without anyone else present in the room and were audio recorded. All interviews were conducted in Dutch and lasted around 60 minutes. All interviews had the following structure: introductory questions about telemonitoring and AI-DSS in general, an explanation of the ARGUS algorithm and follow-up questions about the ARGUS algorithm. The interview guides for patients and cardiologists covered the same overarching topics, with specific questions for clinicians to reflect on the tool in relation to their function as a healthcare specialist and for patients to reflect on the tool in relation to their role as a cardiology patient. No follow-up or repeated interviews took place. The recorded interviews were transcribed verbatim and identifying information was removed. Verbatim transcriptions of interviews and interviewers’ field notes were compared with audio recordings to check for accuracy and were not shared with interviewees for a member’s check. After data analysis and selection of illustrative quotes, JD translated the quotes from Dutch into English for publication and checked them for accuracy and meaning consistency together with the research team.

### Data analysis

The interviews were conducted until meaning saturation was reached, meaning that the interview data were interpreted until a coherent and useful narrative was formulated in relation to the context and the data.^30^ A reflexive thematic analysis was conducted on the transcribed data.^28,29^ Analysis was iterative and reflexive. Throughout, the analytic focus was on developing interpretive themes that captured patterned meanings across the dataset in relation to the research question. Coding was both inductive and deductive, in that it was primarily inductive, while also being informed by concepts from the NIRN Hexagon Model used to formulate the topic list. Codes were examined and systematically reviewed for supporting or conflicting evidence concerning themes and codes. The analysis was performed with software programme NVivo, V.12 and V.15. Themes and initial categories were examined and iteratively developed; main themes in the coding tree were: (1) eHealth and clinical decision-making in general, (2) experiences relevant to integration of the AI-DSS, (3) sex-specificity of the AI-DSS, (4) other (ethical, social and practical) considerations, (5) reasons for or against use, (6) potential impact of the AI-DSS on roles during shared decision-making, (7) responsibility and (8) trust. Two team members (MB and KJ) coded the interviews, performed intercoder reliability checks during the process, resolved coding differences in dialogue to further and deepen the analysis and discussed their findings iteratively within the team.

### Data statement

The data have been presented by means of illustrative quotes, which were carefully selected to represent the arguments presented in the interviews and do justice to the variety of perspectives shown within them.^31^ We use simple frequency descriptors to indicate how many participants expressed a particular view, solely as a form of descriptive support. These counts are not used to infer the importance or significance of themes, but rather to complement illustrative quotations and enhance transparency in reporting patterns across the dataset.^32^ The complete datasets themselves are not publicly available because the individual privacy of the participants could be compromised. This type of interview is deemed sensitive and therefore falls under the protection of the General Data Protection Regulation (article 9).

### Patient and public involvement

This study is part of a broader research project called ARGUS; as reported in a previously published article,^25^ the ARGUS study recruited patients who were referred by their General Practitioner (GP) to the outpatient clinic of Cardiology Centers of the Netherlands (CCN) because of chest discomfort to study macrovascular disease with cardiac CT and microvascular disease with cardiac stress MRI. As part of the ARGUS study’s questionnaire, participants were asked if the researchers could store their contact details for follow-up research questions. Interview study participants were recruited from this population. Participants from the outpatient clinic of CCN were involved in the design of the ARGUS study as a whole and evaluation of the study protocol. Participants received information on the study at a summary level during enrolment, and after the study is completed, they are asked to participate in the evaluation of the study. To prevent role conflicts and ensure the reliability of the results, participants were not directly involved in the study design of the interviews; the interview study therefore served as a support of the Patient and Public Involvement and Engagement (PPIE) approach in the broader project by mainly providing accurate incorporation of and engagement with patients’ perspectives and experiences into the design and conduct of the research on the AI-DSS.^33^

## Results

### Theme 1: Attitudes towards AI-DSSs and the need for a sex-specific AI-DSS

First, we asked participants about their experiences with digital health. Most patients reported a lack of experience with home monitoring, DSSs or medical AI (12/20 PTs), yet some had experience with digital systems in care or research (6/20 PTs) and few because of their occupation (2/20 PTs). All interviewed healthcare providers, on the other hand, had prior experience with eHealth, DSSs or medical AI (11/11 HCPs). Patients’ largely limited experiential grounding did not translate into disengagement: overall patients were interested in the use of AI-DSSs in healthcare (11/20 PTs) or even very positive about its potential application (5/20 PTs), few were undecided (3/20 PTs) and one patient did not consider AI-DSSs in the care context to be valuable (1/20 PTs). Healthcare providers, too, were interested in the use of AI-DSSs (7/11 HCPs) or were very enthusiastic (2/11 HCPs), and a minority were sceptical about the benefits of these systems (2/11 HCPs). These patterns suggest that ‘experience’ with digital health technologies does not directly translate into acceptance or rejection. Instead, attitudes appeared shaped by differing interpretative frames: patients often spoke from a standpoint of anticipated benefit, hope or imagined usefulness, whereas providers drew more directly on situated clinical exposure. When discussing the sex differences in cardiovascular disease and the potential value of the ARGUS AI-DSS for identifying whether men or women need additional diagnostics, few healthcare providers were very positive (3/11 HCPs). Some healthcare providers acknowledged that symptoms differ between men and women but were not convinced a sex-stratified algorithm was needed in the diagnostic process (3/11 HCPs; table 2, quote 1A). Most patients were enthusiastic about the possibility of an AI-DSS to differentiate between men and women, either because their own experience with medically unacknowledged symptoms that turned out to be a cardiovascular disease (3/20 PTs, all female; table 2, quote 1B) or because they considered the goal to improve the accuracy of diagnostic and care important in general (12/20 PTs; table 2, quote 1C). One patient considered it unnecessary to differentiate based on sex in diagnostics.

**Table 2:** Illustrative quotes for theme 1

	Subtheme	Quote
	*The need for a sex-specific AI-DSS*	
1A	HCP unsure about the need for a sex-specific AI-DSS	Of course, a women experiences, well, we know from a lot of studies that complaints present themselves differently in women than in men. I believe this is true and you also see this in practice. But I think you are an all-round caregiver. So, both for men and for women. Which is why I do not understand completely why we should have a tool specifically for women. (HCP2)
1B	The need for a sex-specific AI-DSS from female PT perspective	Well, I think [a sex-specific AI-DSS] is really necessary because I‘ve experienced a lot of misery myself. (…) I think I’ve had about five different diagnoses. I’ve been admitted to the hospital three times for a week each time. You’re really taken away with sirens blaring, and then finally you’re outside, still not knowing what you have. (PT3)
1C	The need for a sex-specific AI-DSS to differentiate between female or male complaints from PT perspective	I think it’s good (…) to also distinguish between men and women, so that it becomes clearer in the doctor’s mind, because I still think it’s highly unclear. What are women’s complaints and what are men’s complaints, or should I not call it that? (PT16)

AI, artificial intelligence; DSS, decision support system; HCP, healthcare professional; PT, patient.

### Theme 2: Anticipated benefits of and concerns about the ARGUS AI-DSS

Following the discussion about the functioning and foreseen use of the ARGUS AI-DSS, respondents articulated various anticipated benefits ranging from improved diagnostic processes to potential reassurance. An often-mentioned benefit of the AI-DSS was that it could provide clarification or verification of the physician’s analysis (12/20 PTs, 8/11 HCPs; table 3, quote 2A), suggesting that participants conceptualised the AI-DSS less as an autonomous decision-maker and more as a supportive tool within diagnostic reasoning. Another reason for using the AI-DSS included the potential improvement of the accuracy and efficiency of the diagnostic process, as the ARGUS AI-DSS may detect some cases that otherwise may be overlooked (12/20 PTs, 5/11 HCPs; table 3, quote 2B); this reflects an underlying assumption that diagnostic processes are inherently vulnerable to omission errors, and that algorithmic support may function as a safety net to counter human fallibility. Further, many respondents hoped that the AI-DSS could improve efficiency in terms of cost-effectiveness: reducing time spent on diagnostics while maintaining accuracy when applying the AI-DSS was seen as a benefit and a reason for using it (8/20 PTs, 6/11 HCPs; table 3, quote 2C). Some also argued that the ARGUS AI-DSS may detect abnormalities in an early stage, enabling follow-up or treatment that prevents future acute care and related costs (6/20 PTs, 1/11 HCPs; table 3, quote 2D). Finally, when discussing potential benefits of the AI-DSS, several respondents alluded to reducing the patient burden in terms of less burdensome diagnostics, less travelling and less time investments (5/20 PTs, 1/11 HCPs; table 3, quote 2E).

**Table 3:** Illustrative quotes for theme 2

	Subtheme	Quote
	*Potential benefits*	
2A	Clarification or verification of the clinician’s judgement	Look, of course you always look critically at each patient, but I think it’s helpful to have an algorithm to supplement your judgment, and it can support you a bit in your decision-making. (HCP2)
2B	Improved diagnostics	It sounds great, especially because they design a tool that minimizes the chances of missing something. I believe that it works, as the tool is so comprehensive that it significantly reduces the likelihood of missing things compared to the current situation. (PT15)
2C	Improve efficiency	If I can work much more efficiently because of it. I think that’s the main thing. So, that I know I’m not treating those patients worse but that I spend half the time on it. Then I would start working with it immediately. (HCP6)
2D	Prevention	I think it can play a role in an early analysis of problems because it, uh, well…. Yes, it’s not so burdensome. So I think you can also use it at an earlier stage and more often, at a lower cost. (PT5)
2E	Less expensive tests and diminish the patient burden	Just because it will save on the CT and MRI scans. Yes, they are expensive tests, maybe time-consuming too. And I personally didn’t enjoy being in the MRI machine either. (PT17)
	*Concerns*	
2F	(In)sufficient reassurance	I do understand that (….) when you have complaints, you’re still inclined to go through that scan, even though the algorithm says, ‘you can go home with peace of mind,’ you still prefer to go through such a scan just to be sure, to rule out anything. (PT13)
2G	Lack of time, the tool may only be beneficial for cases in which there is doubt	The only reason I can think of [for not using the tool] is of course lack of time. You must type in all kinds of things when using the tool. (Yet,) sometimes it goes very quickly at the hospital. (…) (That is,) sometimes you have a high-risk patient who is obese, has diabetes and smokes, then I am finished after two minutes, I don’t need an algorithm. I would not want to fill in all kinds of things. No. And, on the other hand, sometimes I see a clear low-risk patient who is very young, and then I will not use the tool, as it can add fear or would confirm a minor complaint. (HCP7)
2H	Available alternatives	I didn’t know the ARGUS AI-DSS. But (…) I think, sorry to say, it won’t add much value because we already have a validated heart score. Which is a similar algorithm. (HCP 9)

AI, artificial intelligence; DSS, decision support system; HCP, healthcare professional; PT, patient.

Along with these benefits, respondents also mentioned concerns about the value and use of the AI-DSS. Many worried that patients with symptoms would prefer to undergo a scan in order to be reassured that there is no cardiovascular disease underlying their symptoms, regardless of the algorithm’s recommendation (10/20 PTs, 7/11 HCPs; table 3, quote 2F). Healthcare providers also raised concerns about the feasibility and workflow integration of this tool. Some questioned whether they would have sufficient time to meaningfully engage with the system in routine practice and suggested they would only use it in cases where they would experience medical uncertainty (5/11 HCPs; table 3, quote 2G), illustrating that adoption of digital technology is both shaped by its perceived utility and by organisational constraints in clinical contexts. One healthcare provider considered another available algorithm to be an alternative for this new algorithm (table 3, quote 2H), suggesting that alternative tools already fulfil a similar role and thereby questioning the necessity of the ARGUS AI-DSS.

### Theme 3: The impact of the AI-DSS on roles and responsibilities in the clinical decision-making process

The interviews revealed multiple, and at times inconsistent, perspectives on how the AI-DSS affects the roles and responsibilities of healthcare providers, patients, and developers in clinical decision-making. Overall, patients agreed that the algorithm cannot fully replace the healthcare provider (9/20 PTs; table 4, quote 3A), even if some respondents thought that HCPs and AI-DSSs reason according to similar decision-rules (2/20 PTs). At the same time, a more nuanced reconfiguration of professional roles was envisioned by some patients and cardiologists, who anticipated the partial substitution of cardiologists by other healthcare professionals who would rely on the AI-DSS, such as residents or general practitioners (4/20 PTs, 5/11 HCPs; table 4, quote 3B). Others argued that the role of the cardiologist will remain the same whether HCPs use the AI-DSS or not, because they perceived the AI-DSS as one of many information sources at the clinician’s disposal or because they stressed the need for shared decision-making (17/20 PTs, 7/11 HCPs; table 4, quote 3C). When discussing the clincian’s role in shared decision-making, many respondents emphasised that the physicians’ role includes having a conversation about the findings and comforting patients, and that they should not blindly follow the algorithm (12/20 PTs, 10/11 HCPs). Moreover, few respondents reckoned that HCPs have a trained gut-feeling and, based on their intuitions, physicians should be allowed to overrule the AI-DSS (3/20 PTs, 2/11 HCPs; table 4, quote 3D). These viewpoints underscore the continued legitimacy attributed to embodied, experiential knowledge of clinicians, regardless of the presence of digital decision support tools. Some HCPs also stressed that patients’ needs and preferences should be accommodated, also if it means conducting extra diagnostic tests to comfort them or overcome some of their fears (4/11 HCPs; table 4, quote 3E), thereby extending medical responsibility beyond strictly evaluating the available evidence or algorithm-driven outcomes. When discussing who is responsible for the clinical decision when the ARGUS AI-DSS is used, most argued that the HCP should remain fully responsible (11/20 PTs, 10/11 HCPs; table 4, quote 3F). Yet, this coexisted with notable unease among some clinicians regarding the fairness and feasibility of assigning full responsibility to them in contexts where algorithmic systems are neither fully transparent nor perfectly accurate. Few patients argued that, while HCPs are responsible for false AI outcomes, they themselves carry final responsibility for the medical decision (3/20 PTs; table 4, quote 3G), signalling the active role that patients want or assume in shared decision-making. Others suggested a shared responsibility of HCPs and tech-developers (4/20 PTs), where the HCP is responsible for the use and interpretation of the tool, and the developer carries the responsibility for the algorithm and its outcomes (table 4, quote 3H). More rarely, patients attributed responsibility to the algorithm itself or to its developers as primary sources for accountability. That is, one patient mentioned that the ARGUS system itself should carry the responsibility and one patient argued that the full responsibility lies with the technology developer. Some patients also referred to shared responsibility between patients and HCPs, explaining that patients remain responsible for providing accurate information (7/20 PTs). HCPs mostly referred to the role of patients in terms of shared-decision-making, which was not perceived to be influenced by the AI-DSS, but did not describe this explicitly in terms of responsibility (7/11 HCPs; table 4, quote 3I).

**Table 4:** Illustrative quotes theme 3

	Subtheme	Quote
*The impact of the AI-DSS on clinical roles and responsibilities*		
3A	No full replacement of face-to-face consultations	If the contact with the specialist or the general practitioner is no longer face-to-face but just a printout from a computer, based on which you then…. yes, that may be helpful but not on its own. Sometimes you get the feeling that soon no doctor will be needed anymore because everything can be done based on data, but of course, it can’t be like that. (PT14)
3B	The ARGUS AI-DSS: a tool for other HCPs	Yes, honestly, I don’t know what I [as a highly trained, expensive cardiologist] am doing there [managing the ARGUS AI-DSS.] You don’t need a cardiologist for that. (…) I’m not the one who needs to stand there. It can also be a professional who orders all the tests, checks the boxes, and does the things. I’m not needed for that. (HCP10)
3C	The cardiologist’s role does not change, yet sources of information increase	The cardiologist’s role doesn’t change, but their sources of information increase. They have more resources to make a better diagnosis and thus make a decision, but their role remains the same. (PT10)
3D	The clinician can overrule the AI-DSS based on intuition	That’s why I asked, do you see the doctor then? I think it’s just algorithms…. a good doctor also has their intuition, or they can see someone or hear something, or yeah. I think as a supplement, look, in the end, the doctor decides (…) I think it’s a very valuable source of information. Yeah. I don’t think it’s wise that if the algorithm says it’s not necessary and the doctor thinks it is, that they don’t do it. I believe the doctor should have the final say. (PT10)
3E	Dealing with fear of the patient and the need for reassurance	But look, because I always believe that ultimately it’s about the patient, and if they need reassurance or closure regarding a certain complaint or situation, I often go along with that. Of course, I advise, and yes…. I do think that if a patient says, ‘‘I notice that this really preoccupies me and I’m afraid of it,’’ I always discuss beforehand: do you truly think that this examination will reassure you? (HCP2)
3F	HCPs should remain responsible, also when using AI-DSSs	I think the decision is always made by the cardiologist. So I also believe that the cardiologist is responsible in that regard. Unless you’re going to make very strict directives. So, if it’s said, okay, if the score is above 6, then we will do this. If the score is below 6, then we will do that. And by doing so, you are essentially tying the hands of the cardiologist, and I don’t think he would still be responsible because then you’re being forced into a straitjacket. And the question is whether that is desirable. I personally think not. But essentially, I believe the healthcare provider is always responsible for what they perform and what they consider in the decision-making process. Just like with any examination, interpreting it and the final decision will be, in my opinion, the responsibility of the healthcare provider because they have a doctor-patient relationship. So I wonder if you…. I think, in any case, a medical examination or test itself never has a doctor-patient relationship, so I wonder if that is legally or even possible to take that step. Honestly, I don’t think so. (HCP8)
3G	HCPs are responsible for algorithmic mistakes, but the patient is responsible for the final decision	I think the doctor has the responsibility [when the algorithm makes a wrong prediction]. That also seems very wise to me, to give them the ultimate responsibility so they stay alert and don’t just see a note and then think, okay, we’re done. Then we have a little chat and send the bill. No, that’s not right, and besides, they also took an oath, so to speak, and you know, as a patient, you have to place some trust in them. So I would definitely assign that to the doctor, yes. (…)[However, when it comes to who is leading in the decision, then] no, I would ultimately say the final responsibility lies with the patient*,* and you can only make a decision if you trust that doctor. (PT10)
3H	Shared responsibility: HCP and tech developer	Yes, I think the developer [is responsible for] the outcome. But perhaps the interpretation is ultimately the cardiologist’s. (PT18)
3I	Shared responsibility: HCP and patient	I think it’s a collaboration between the doctor and yourself. Because you have to provide your own information about your lifestyle and so on. And how does the doctor know whether everything you record about your blood pressure is the truth*?* (PT16)

AI, artificial intelligence; DSS, decision support system; HCP, healthcare professional; PT, patient.

### Theme 4: Conditions for implementation

Based on the anticipated benefits, roles and concerns associated with the ARGUS algorithm, several preconditions were mentioned that should be in place for the proper use of such an AI-DSS. Rather than functioning as isolated technical requirements, these conditions referred to broader expectations about safety, professional oversight, usability and ethical governance of AI within healthcare.

A frequently-mentioned condition for adoption was the technological soundness of the AI-DSS in terms of robustness, validation and reliability (6/20 PTs, 9/11 HCPs). Clinicians specifically indicated the importance of having confidence in the consistency and accuracy of the tool across patient populations relevant to routine care. Another condition for use that many respondents emphasised, was the necessity of a human interpreting AI outcomes or scores (table 5, quote 4A), or a human-in-the-loop, meaning that a healthcare professional should always check the outcome of an AI-DSS and be able to overrule the algorithm’s advice (9/20 PTs, 5/11 HCPs; table 5, quote 4B). Furthermore, one healthcare professional believed that effective and quick implementation requires wide user adoption (1/11 HCPs; table 5, quote 4C) and one patient mentioned that AI systems should be regularly updated to take new or unknown pathologies into account (table 5, quote 4D). This suggests that AI systems in healthcare should be maintained as dynamic tools subject to ongoing validation and refinement rather than static implementation. Many cardiologists also mentioned that it was necessary for the AI-DSS to be easy to use (6/11 HCPs; table 5, quote 4E), highlighting the importance of workflow compatibility and minimal disruption to existing diagnostic processes. Finally, some patients thought that the drive to reduce healthcare costs should not be at the expense of good quality care (5/20 PTs; table 5, quote 4F) and one patient mentioned the importance of including ethicists in the model development (table 5, quote 4G), indicating awareness of the broader dimensions of AI implementation in healthcare beyond technical and clinical validation alone.

**Table 5:** Illustrative quotes theme 4

	Subtheme	Quote
*Conditions for implementation*		
4A	AI-DSS should be viewed as a score generator that cardiologist needs to interpret	It has been validated in a specific patient group, and it does not always apply to the patient directly opposite you. And it’s statistics, for example with a VAS score. If you’re 65 or older, you get a point, and then your score becomes a bit higher, and you need blood thinners; otherwise, you don’t. Yeah, and if you’re 64, then you wouldn’t need blood thinners. So, that kind of decision-making will always be done by the cardiologist. (HCP9)
4B	Human in the loop where HCP checks AI outcomes and overrules the system when deemed appropriate	It remains the case that I think that if you yourself, if you have a gut feeling or sense that something’s not right, would always overrule the system. (HCP 2)
4C	AI-DSS needs to be widely adopted in clinical practice	Look, you all have to do it together, and you just have to do it. And you shouldn’t complain about it. And after a year, you’ll see, well, how does the algorithm need to be adjusted, because it’s also a learning algorithm. And then we won’t just do it in Utrecht, but we’ll do it in all hospitals in the Netherlands. Well, then it will happen quickly. Very nice. And I… but all need to use [the AI-DSS] in the hospital. Because if there are some who do not participate, it undermines the whole system. (HCP 10)
4D	Regularly updated to take new or unknown pathologies into account	Suppose someone had COVID-19 without you knowing and as a result, developed heart problems. That’s not yet included in those algorithms because it’s new, and with all these new variants, new pathologies can also emerge that are not yet known, like recently with thrombosis, which turns out to be a very specific condition caused by AstraZeneca. (Interviewer: So, an algorithm must always….) Stay in motion, yes, be adapted. But it will always lag behind new developments. That’s why a specialist is always needed*.* (PT14)
4E	Easy to use	Accessibility and simplicity. The simpler the model is, the better. It really needs to be integrated into clinical care practices, preferably without the doctor having to enter six or seven other variables. It should be integrated into the electronic patient record. Those are important factors because everyone is also heavily burdened with administrative tasks. And if you — for the use of the model — would need to open a website, find the right one, and enter eight variables that you’ve already entered into the EPR, it just won’t work. (HCP1)
4F	Quality of care should be primary reason for use, not cost reduction	So, a doctor, a cardiologist, should not be driven by, so to speak, being forced by the hospital to start using this for cost-saving reasons and therefore just to do it. That is the main risk factor. The pressure becomes so great, so to speak, the costs… that it just gets taken for granted and is used. (PT1)
4G	Include ethicists and discuss ethical questions AI models raise	Because that seems almost conditional if you're going to work with this kind of algorithms, that you also have an ethical discussion about it. And that you train doctors in this as well. Yes, they obviously receive medical ethics training, I assume, but that is becoming increasingly important, especially as it takes on a new form through these digital possibilities we now have, yes, it will also raise different medical ethical questions. (PT10)

AI, artificial intelligence; DSS, decision support system; HCP, healthcare professional; PT, patient.

### Theme 5: The relevance of trust when considering AI-DSS use

Trust emerged as a central and multilayered topic across the interviews. Rather than only considering the trustworthiness of the system, the decision to trust the AI-DSS was described as a dynamic choice depending on how well the model’s outcomes align with patients’ illness experiences, clinicians’ intuitions and judgements, the extent to which respondents have positive personal experiences with the model, and whether colleagues are already using it.

One patient, for example, mentioned that a combination of knowing what information is taken into account in the algorithm and being on the same wavelength as the HCP would build trust in the decision not to perform an additional scan (table 6, quote 5A). This reflects an important relational dimension of trust, particularly when decising against additional diagnostic tests. A couple of patients also discussed that they trust scientific innovations and data-driven models in general and would therefore also trust AI-DSSs (6/20 PTs; table 6; quote 5B), suggesting that trust in AI-DSSs may, for some, be grounded in a more general confidence in biomedical progress and quantitative models. Across both patients and healthcare providers, however, trust was more commonly framed as conditional and contingent, dependent on a set of personal quality and use criteria.

**Table 6:** Illustrative quotes theme 5

	Subtheme	Quote
	*Trust and conditions for trust in the AI-DSS*	
5A	(Partial) explainability of the model and synergy with HCP could build trust	Yes, if there is insight into which information—or a large part of it—an algorithm takes into account, then that could potentially build trust. (…) If I speak for myself, I would say that if I trust the data coming from such an algorithm and the doctor sitting across from you is also somewhat on your wavelength—because of course, it can also clash a lot—well, I’m putting it very black and white now, but I mean, characters don’t always have to match. I think it’s easier to accept that and say, well, fine, just leave that scan because I do trust it. If you still have doubts, then you should also mention that. (PT16)
5B	Trust in the scientific process	It was conceived and carefully weighed by scientists. And so I trust it. (PT17)
5C	Validity and inclusion in clinical guidelines provides confidence	Once everything has been properly validated in the study, we have confidence in it. And if it gets included in the [clinical] guidelines. (HCP9)
5D	Proof of patient benefit and transparency regarding the development process are conditions for trust	I want to understand how the algorithm is developed, that’s one. And two, I want to see proof and validation of the algorithm’s positive impact on decision-making processes concerning all kinds of [patient] cohorts. So [I want to know] that patients will have better results if you use the algorithm. (HCP6)
5E	Trust is heightened when the AI-DSS is widely adopted by colleagues	It would also be nice if it is embraced and used throughout the Netherlands; that also provides reassurance. (HCP7)
5F	Oversight by patient organisations to stimulate trust in the clinical adoption	At the moment an algorithm is fully developed and super reliable, I think there still needs to be oversight from patient organisations or a similar party to ensure that an algorithm doesn’t become the sole decision-maker. (PT8)
5G	Trusted by the specialist leads to patient trust	If the doctor has trust in it, yes, I think you should listen to that specialist. Unless he or she says ‘’I don’t trust it, I think that…’’ (PT13)
5H	Personal experience with the AI-DSS shows how much you can trust it	How much trust you place in a specific model always matters. Over time, you’ll gain experience with how the model performs, the outcomes it produces, and the choices you’ll make. Eventually, you’ll have a clear sense of whether or not you believe it is a reliable model. (HCP2)
5I	Alignment with the patient’s intuitions and experiences of symptoms matters for patient trust	I also think about how you, at that moment, when you have a lot of complaints, then…. That’s how it works for me anyway. Then you also think, there must be something, right? So they should keep looking because this can’t be right. Because if that were the case, I wouldn’t have much trust in it. But when the complaints also start to decrease again, then yes, you do have trust, well, indeed. (PT2)
5J	Involvement of health insurance decreases trust	If I had the feeling that the health insurer is behind this, I would trust it less. It could still be a very good tool, by the way, but I wouldn’t find that comfortable. (PT10)

AI, artificial intelligence; DSS, decision support system; HCP, healthcare professional; PT, patient.

Across the interviews, validity was consistently named as a condition for trusting an outcome (14/20 PTs, 10/11 HCPs), as many respondents believed that the algorithm must demonstrate robust performance and undergo external validation before being considered trustworthy in clinical decision-making. Some explicitly mentioned that they associated validity with having tangible proof that the AI-DSS provides patient benefit and that the system can be included in clinical guidelines (table 6, quotes 5C and 5D). Trust of other stakeholders or colleagues in the AI-DSS was also often mentioned as a confirmation of its value and therefore a reason to trust its outcome. Some HCPs, for example, found it important that the algorithm is trusted and used by colleagues (5/11 HCPs), and that they would themselves trust its use (table 6, quote 5E). Others mentioned that oversight by a patient organisation would be important for them to trust its application (2/20 PTs, 1/11 HCPs; table 6, quote 5F). A few patients uttered that they would trust the AI-DSS, as long as the doctor or specialist trusts it too (8/20 PTs; table 6, quote 5G). Interestingly, a few patients reversed this argument and said that the ARGUS AI-DSS could support trusting the diagnosis of the physician and referred to trust in terms of confirmation (3/20 PTs). Another trust-fostering aspect that was mentioned was the HCP having had positive personal experience working with the AI-DSS (5/20 PTs, 3/11 HCPs; table 6, quote 5H), indicating that familiarity can contribute to building trust and acceptance of AI-DSSs. Moreover, trust in the technology was often associated with the extent to which it aligned with participants’ own intuitions and perceptions of their health status (2/20 PTs; table 6, quote 5I); this ‘phenomenological alignment’ suggests that confirmation of pre-existing expectations rather than challenging them shapes initial trust in such tools. Conversely, a factor that was perceived to obstruct trust in the AI-DSS was the involvement of health insurances in its development and implementation (2/20 PTs, 1/11 HCPs; table 6, quote 5J). It reflects a sensitivity to perceived conflicts of interest in healthcare technology, which may undermine confidence in the neutrality and patient-centredness of the system.

## Discussion

We conducted a qualitative interview study to explore how specific systems can transition from research settings to practice and identify which socioethical considerations are important during the transition. We specifically focused on how well the ARGUS algorithm aligns with the clinical environment and which ethically relevant considerations are deemed important by its prospective end users. Such user-centric studies are tantamount to responsibly crossing the chasm^34^ to medical practice and ensuring that AI meets clinical values and needs.^35^ Our study integrated the perspectives of clinicians and patients and also marks the first qualitative analysis of an AI-DSS aimed at *excluding* cardiac disease, suggesting that its insights could be valuable in other contexts, especially as disease exclusion becomes an emerging focus in AI development targeted at cardiology.^36^

Notably, similar to other qualitative studies on the clinical value of AI in cardiology and other medical specialties,^37–41^ respondents in our study generally recognised the added value of using an AI-DSS targeted at excluding CAD as a supplement to clinical judgement. When discussing the anticipated benefits, both cardiologists and patients seemed to prioritise increased accuracy and efficiency, while a relief of the patient burden was mentioned less frequently. This can be understood in two ways: first, respondents’ views might reflect wider conversations that highlight accuracy and efficiency as key advantages of medical AI.^42–45^ Second, easing patients’ burdens was often seen as secondary to the importance of ascertaining if the symptoms patients experience indicate serious cardiac conditions. During the interviews, respondents highlighted several concrete practice-driven aspects that need further attention in the ARGUS development and implementation process, which may also apply to integrating other AI-DSSs into the clinic. One key concern raised by many cardiologists and patients—particularly aimed at AI systems focused on disease exclusion—is whether a low likelihood of CAD provides sufficient reassurance to patients; many may still choose to undergo a scan, regardless of the algorithm’s recommendation. As other qualitative studies show, distrust in AI recommendations is not uncommon among patients,^41^ and patients’ individual circumstances and values are essential in collaboratively deciding on the best course of action.^46^ Some other concrete concerns mentioned in the interviews were more group-specific. For instance, as in other qualitative studies,^24,40^ HCPs cited practical reasons as barriers to using this algorithm: they were concerned that managing the system would consume valuable time and that it might not deliver on its promises of efficiency. Additionally, they concentrated on the algorithm’s usability, particularly its ease of use and whether it has been widely adopted. Interestingly, this creates a paradox: someone must use the algorithm first before others can evaluate its usability and decide whether they are inclined to use it. Patients, in contrast, more frequently expressed their need for reassurance that AI systems like the ARGUS AI-DSS are not designed solely to reduce healthcare costs or to serve health insurers. They emphasise an important aspect also noted in the literature regarding the cost-effectiveness of medical AI: patient outcomes or the quality of care instead of cost-effectiveness should be the primary focus of medical AI.^47^ Nevertheless, while commercial incentives for the development of AI should not be the main driver of innovation, cost-effectiveness acts as a vital element to assess development feasibility and should be considered when developing AI, as Gomez Rossi *et al* indicate. Moreover, the interviews revealed a connection between this use case and broader ethical concerns and unresolved questions, indicating that this study can provide valuable insights into how AI ethics discussions apply to and are made specific in the clinical application of specific AI systems. Key connections between the interviews and ethical literature include the following topics: (1) the need for sex-specific AI-DSSs in cardiology, (2) exploring shared responsibility for AI in clinical practice and (3) over-ruling the AI system in cases of human-AI disagreement. In the next sections, we will further discuss these in connection to arguments made in the academic literature.

### The need for sex-specific AI-DSSs in cardiology

Recently, there has been a greater focus on the differences between the ways cardiac conditions present in male and female patients. Taking these differences into account is considered crucial for fair AI design in cardiology, and many argue that a greater emphasis should be placed on developing suitable systems for specific sexes^48–51^; the ARGUS AI-DSS is a good example in which that is taken to heart. In this study, however, only a small number of cardiologists were very optimistic about developing a sex-specific AI system for excluding CAD; some even showed a lack of understanding regarding the necessity of such a tool. Their attitudes towards a sex-specific tool may indicate that, before deployment, it is important to further discuss (1) the fact that AI will not resolve differences between men and women without actively developing specific sex-specific models and (2) the existing bias towards men in cardiology practice with current practitioners; and critically examine how such an AI-DSS can play a role in diminishing such harmful biases. Patients, on the other hand, were generally enthusiastic about the development of a sex-specific system, especially women who had experienced unacknowledged symptoms. These experiences are echoed in Frich *et al*’s qualitative study,^52^ in which it is illustrated that healthcare practitioners often view CAD primarily as a male condition, creating significant obstacles for women in terms of timely and adequate diagnosis and treatment. Other studies, such as the one *Hao et al*, also confirm that women often receive worse diagnoses and treatments for the same disease.^53^ Acknowledging the sex-specific ways in which cardiac conditions are presented is therefore essential, yet, as pointed out in other studies focusing on sex-specific AI in cardiology,^48,54^ the development and implementation of fair sex-specific algorithms is impeded by several challenges that should receive further attention, also in the case of the ARGUS AI-DSS. For instance, gaps in medical knowledge about potential differences in causal mechanisms underlying cardiac conditions and the underrepresentation of women in training data remain significant obstacles for sex-specific advancements algorithms.^48^ Furthermore, it should be considered whether differentiating by sex is always desirable and sufficient, or whether other patient characteristics should also be made available and taken into account; the effects of ethnicity or belonging to a disadvantaged socioeconomic group on cardiac problems, for instance, are not well known and should be investigated.

### Exploring shared responsibility for AI in clinical practice

As reported in other empirical studies on medical AI,^31,55,56^ most respondents in this study believed healthcare professionals should be fully responsible for the use of AI in medical decision-making. Nevertheless, some cardiologists were hesitant to assume responsibility for AI due to opaque algorithms and the risk of algorithmic errors. The lack of transparency and control over errors in black-box AI is a broader concern in academic literature^57^ and could create responsibility gaps.^58,59^ To remedy some of their concerns, Bezemer *et al* argue that healthcare professionals should initiate and guide the development and implementation of AI-DSSs to enable them to become responsible users.^60^ An alternative that patients mostly brought up in this study is shared responsibility—either between patients and HCPs or HCPs and AI developers—and may be worth exploring. The possibility of shared responsibility, especially between HCPs and AI developers, has been suggested in numerous instances as a way forward.^61,62^ Whitby, for example, maintains that shared responsibility between healthcare professionals and AI developers can help to prevent algorithmic errors.^62^ Nevertheless, few authors have elaborated on how responsibility should be allocated between clinicians and technologists,^57,62^ which is essential to explore the desirability of this possibility further. Shared responsibility between HCPs and patients has so far seemed to have received less explicit consideration. It has, however, been argued that while actively involving patients is conditional to valuable AI, assigning responsibility or even blame to patients should be approached carefully.^59,63^ Furthermore, a challenge associated with shared responsibility is that it may not be as straightforward as respondents and authors who have examined this possibility have anticipated. That is, many different agents can be involved in the development and implementation of an AI-DSS—not just AI developers and end users. For example, Bleher and Braun outline the range of potential agents involved: ‘‘the system’s developers; the providers of technologies, such as software or tech companies; the system’s operators, such as hospitals or other public health institutions; all data subjects who enter or have previously entered their data into the system—all these and others may be deemed agents in the context of AI-(…)DSS[s].’’^64^ Shared responsibility may therefore necessitate a comprehensive analysis of the agents involved in each aspect of the development or use of the AI-DSS, and a clearer understanding of the implications of assuming responsibility for a specific segment of its application.

### Overruling the AI system in cases of human-AI disagreement

Related to the necessity for HCPs to bear the responsibility for AI, respondents frequently underscored the importance of HCPs keeping a certain level of control over AI’s role in medical decision-making. That is, several cardiologists and patients thought that HCPs should always check AI outcomes and overrule these when deemed necessary. In the academic literature, the necessity for healthcare professionals to review and override AI is frequently discussed in connection with human-AI disagreement, emphasising the importance of a human in the loop or maintaining meaningful human control.^65,66^ Interestingly, patients also specifically stated that their trust and acceptance were based on the combination of a valid model, as well as the amount of experience of the healthcare professional, and whether they would maintain professional oversight. At the same time, while human oversight offers many advantages and should not be overlooked, users need to be cautious about dismissing AI too hastily and being overly confident in their own expertise. Studies have shown that confirmation bias can be just as dangerous as automation bias, indicating that practitioners will often follow their own judgement and intuition rather than the AI outcome at the expense of the patient.^67^ This tendency was also observed in the interviews, where cardiologists mentioned that they would trust or rely on the AI system if it confirmed their own intuitions or, in the case of patients, feelings of being ill or not. Some cardiologists even mentioned that they would not need an AI-DSS when they were certain of someone’s risk. Yet, intuitions can, for example, be founded on unwanted biases towards the ways in which cardiac diseases manifest in specific patients; relying on these too heavily can cause harm. A question for the implementation of AI-DSSs, including the ARGUS AI-DSS, is, therefore, how to balance and train users to distinguish between AI outcomes that should be followed and those that should be overruled. This question also merits further analysis of how AI and the tacit aspects of the medical profession interact and should be weighed, especially since trained intuitions and AI outcomes can be based on different explicit and implicit evidence bases—though the development of AI can potentially also help convert some of the practitioners’ tacit knowledge into structured, data-driven insights and require harmonising both evidence bases.^68^

### Future outlook, strengths and weaknesses

To be able to fully support the adoption of AI-DSSs during shared decision-making, this study confirms the importance of studying the ‘need‘ and ‘fit’ domains of the NIRN Hexagon Tool by interviewing prospective end users; in this case, healthcare professionals and patients. As ethical implementation is intertwined with development, our research also corroborates the importance of multidisciplinary algorithm-development teams that include healthcare professionals and patients. At the same time, as implementation models specify, there are more aspects to the successful implementation of AI that go beyond the scope of this study. For example, according to the implementation Hexagon of NIRN, implementation is divided into six domains: three implementating site indicators (‘need’, ‘fit’ and ‘capacity’) and three program indicators (‘evidence’, ‘usability’ and ‘supports’).^27^ System developers and implementers, and other stakeholders, should also pay further attention to the other domains, not only looking at the characteristics of the innovation but also focusing on, for example, (early) adopters and local culture. This way, implementation will ideally be part of a continuous, ethically sensitive sociotechnical improvement cycle that can be aligned with the technical software life cycle norms.

Our results should be viewed in the context of the following limitations and directions for future research. First, our findings are context-specific and are not intended to be statistically generalisable; rather, they provide transferable insights into the responsible transition of AI-DSSs from research settings to clinical practice, specifically the implementation of digital health tools in cardiology settings. Second, it should be considered that the data were collected in 2020 and 2021; even though this is the case, the implementation process of the ARGUS AI-DSS is ongoing, and the socioethical considerations mentioned then are still at the forefront of both broader AI ethics discussions and the integration process of this AI-DSS. Third, any qualitative interview study is prone to interviewer and researcher bias; a different interviewer could have focused their attention on different aspects of respondents’ answers, and grouped the codes and themes differently. Fourth, the ethical aspects raised in this empirical study require further and more detailed normative analysis to gain practical guidance regarding the design and integration of AI-DSSs. Finally, our findings on trust, particularly the practical reasons participants cited for placing trust in the system, warrant further analysis and may contribute to understanding the differences between perceived and actual trustworthiness.^69^

## Data Availability

No data are available.
